# Supplementary material for: Validation of an Academic Self-Attribution Questionnaire for Primary and Secondary School Students: Implications of Gender and Grade
Source: Int J Environ Res Public Health. 2022 May 16;19(10):6045. doi: 10.3390/ijerph19106045 (PMC9141320; doi:10.3390/ijerph19106045)
Supplement: Supplementary file 1 [file ijerph-19-06045-s001.zip › ijerph-1682176-supplementary.pdf]

UNIVERSIDAD DE BURGOS

Campus de Excelencia INTERNACIONAL

Academic Success and Failure Attribution Questionnaire (León and Mendo, 2020)

Mark with an X the **number** that best fits the causes you use as an explanation when in general you pass or fail an exam.

1	2	3	4	5
Nothing Agreement	Some Agreement	Some Agreement	Very Agreement	Totally Agreement

Success: I approve					
I approve because I try hard in class	1	2	3	4	5
I approve because I try hard in class	1	2	3	4	5
I approve because I try hard in class	1	2	3	4	5
I approve because I use some strategy to prepare the exams (I organize, summarize, review, memorize the topics)	1	2	3	4	5
I approve because I'm so smart	1	2	3	4	5
I approve because I have a very good memory	1	2	3	4	5
I approve because I have a lot of talent, that is, I have a lot of natural capacity for studies.	1	2	3	4	5
I pass because I have a calm character and I don't get nervous in exams	1	2	3	4	5
I pass because teachers put easy exams	1	2	3	4	5
I approve because I have good luck	1	2	3	4	5
I approve because my teachers explain the topics very well.	1	2	3	4	5
I approve because my teachers explain the topics very well	1	2	3	4	5
**Failure: I suspend**					
I fail because I try little in class	1	2	3	4	5
I fail because I spend little time preparing for exams	1	2	3	4	5
I fail because I pay little attention in classes	1	2	3	4	5
I fail because I do not use strategies to prepare the exams (I organize, summarize, review, memorize the topics)	1	2	3	4	5
I fail because I'm not very smart	1	2	3	4	5
I fail because I don't have a good memory	1	2	3	4	5
I fail because I have little talent, that is, I have little natural capacity for studies.	1	2	3	4	5

I fail because I have a nervous character and I can't calm down on exams	1	2	3	4	5
I fail because teachers take difficult exams	1	2	3	4	5
I suspend because I'm unlucky	1	2	3	4	5
I fail because my teachers do not explain the topics well.	1	2	3	4	5
I fail because the level of demand in my class is very high.	1	2	3	4	5

ASFAQ Percentiles

		Controllable internals Success	Non-controllable internals Success	Externals Success	Controllable internals Failure	Non-controllable internals Failure	Externals Failure
**N**	**Valid**	562	562	562	562	562	562
	**Missing**	0	0	0	0	0	0
**Percentiles**	**10**	10	7	7	4	4	4
	**20**	12	9	8	4	4	4
	**30**	14	11	9	4	5	5
	**40**	15	12	10	5	6	5
	**50**	16	13	10	6	6	7
	**60**	16	14	11	8	8	7
	**70**	17	15	12	9	10	9
	**80**	18	16	13	12	12	11
	**90**	19	18	14	14	14,7	13
	**100**	20	20	20	20	20	20
